# Editorial: The biochemistry of amyloids in neurodegenerative diseases, volume II

**DOI:** 10.3389/fnins.2023.1236518

**Published:** 2023-06-27

**Authors:** Cláudio M. Gomes, Wolfgang Hoyer, Jinghui Luo

**Affiliations:** ^1^Biosystems & Integrative Sciences Institute, Faculdade de Ciências, Universidade de Lisboa, Lisboa, Portugal; ^2^Departamento de Química e Bioquímica, Faculdade de Ciências, Universidade de Lisboa, Lisboa, Portugal; ^3^Institute of Physical Biology, Heinrich Heine University Düsseldorf, Düsseldorf, Germany; ^4^Institute of Biological Information Processing (IBI-7) and Jülich Center for Structural Biology, Forschungszentrum Jülich, Jülich, Germany; ^5^Department of Biology and Chemistry, Paul Scherrer Institute, Villigen, Switzerland

**Keywords:** protein aggregation, Tau, synuclein, Parkinson's disease, Alzheimer's disease, prion protein, huntingtin (HTT), chaperone

The aggregation of proteins into amyloid fibrils is a key feature of multiple disorders, in particular neurodegenerative diseases such as Alzheimer's disease (AD), Parkinson's disease (PD), Huntington's disease (HD) and prion diseases. The critical role of protein fibrils and oligomeric assemblies for pathogenesis is widely acknowledged, yet many questions remain unanswered, such as the identity and activities of the most relevant aggregate species and the factors that trigger or inhibit their formation. The articles included in this Research Topic and e-book take a close look at the fate of amyloidogenic proteins alon g the pathway from their monomeric states to oligomeric assemblies and seeding-competent amyloid fibrils. A recurring motif is the enormous structural versatility of the involved proteins, which needs to be appreciated in order to elucidate their functions and dysfunctions.

Structural versatility is evident already at the level of protein monomers, which are often intrinsically disordered, ready to structurally adapt to various binding partners. Bisi et al. review experimental and computational studies analyzing the conformations adopted by α-synuclein. They comprehensively describe the effects of various factors on α-synuclein's conformational ensemble, including disease-related mutations, metal ions, lipids, proteins, and small molecules. In order to map the metal binding sites in α-synuclein by NMR, Gonzalez-Garcia et al. combine amide protection factor determination with relaxation and chemical shift perturbation data. Different monovalent and divalent metal ions have variable effects on the different observables, indicating that the binding modes of the metal ions differ, although they all eventually promote α-synuclein aggregation. Further modulators of α-synuclein's properties are posttranslational modifications (PTMs), with N-terminal acetylation as a prominent example. Iyer et al. provide an overview of the effects of N-terminal acetylation on conformation, lipid binding, and amyloid formation of α-synuclein. The authors discuss that N-terminal acetylation might prime the protein for further PTMs which are crucial for α-synuclein function. In the case of protein Tau, an additional layer of versatility is added by the presence of six different protein isoforms, with a connection of certain Tau isoforms to specific tauopathies. Bachmann et al. report how the individual isoforms behave in a cellular context. They find that the isoform affects the extent of axonal sorting as well as cell size and microtubule number, revealing isoform-dependent microtubule dynamics.

Oligomers are candidates for the main pathogenic triggers, with strong evidence in particular for Aβ oligomers in AD. Iliyasu et al. review pathways involved in AD pathogenesis, including oxidative stress, neuroinflammation, and Aβ clearance. Relating to Aβ proteostasis, Figueira et al.  report how different multimers of S100B, an extracellular signaling Ca^2+^-binding protein with holdase-type chaperone activity on Aβ, achieve inhibition of oligomer and fibril formation. Analysis of the chemical kinetics of Aβ aggregation reveals that especially formation of Aβ oligomers by fibril surface-catalyzed secondary nucleation is hampered in presence of S100B multimers. The inhibitory activity depends on the oligomeric state of S100B, with sub-molar potency observed for S100B tetramers. Nazere et al. study the mechanism of cellular uptake of Aβ oligomers. Their data supports a major role of macropinocytosis, depending on lipid rafts and heparan sulfate proteoglycans, and regulated by the GTPase Rac1.

Several articles in this Research Topic address the formation of amyloid fibrils and their potential to act as seeds in the amplification of protein aggregation. Angelli et al. compare the conversion of rabbit and mouse cellular prion protein (PrP^C^) to the scrapie form (PrP^Sc^). Rabbits exhibit a comparatively low propensity for prion infection. In line with this, the authors find a reduced propensity of rabbit PrP^C^ to convert to PrP^Sc^ *in vitro*, independent of the nature of the individual cofactors used to trigger conversion. Closing in on the pool of species that drive the pathogenic process in HD, Schindler et al. describe the detection of small (average length of 200 nm) fibrils of mutant huntingtin in the brains of knock-in HD mice. These small, soluble particles are seeding-competent in a sensitive FRET-based protein amplification assay. The authors conclude that not only non-fibrillar oligomers but also fibrillar structures are part of the pool of soluble species and can contribute independently to the pathogenic process. Whether or not the fibril structure of the seed is maintained during seeding is the topic of a review by Alijanvand et al.. The authors point out the necessity to differentiate between seeding through fibril elongation and seeding through secondary nucleation, i.e., through nucleation of new fibrils from monomer that is facilitated by the presence of existing fibrils. While fibril growth by monomer addition has been shown to maintain the structure of the seed fibril, the available data does not support the same for fibril amplification through secondary nucleation, where the structure of the new fibril seems to be determined rather by other factors such as the solution conditions. As amyloid fibrils interact differently with ligands than monomers do, Roldán-Martín et al. elaborate on the interactions of an S-shaped Aβ fibril polymorph with metal ions Cu^2+^ and Al^3+^. Applying structural prediction of metal binding sites, protein-ligand docking, and molecular dynamics simulations, the authors find the metal ions bind to distinct sites, i.e., the N-terminus vs. residues Glu22 and Asp23, with opposed consequences for fibril stability. Amyloid fibrils can also be modified and stabilized by dityrosine (DiY) formation, however, the consequences of DiY formation for aggregation and toxicity are not fully understood. Maina et al. review the current literature on the effect of DiY formation on Aβ and Tau assembly and (dys)function. They point out the variability of the results obtained thus far, describe the complexity inherent to DiY modification of aggregating proteins, and discuss the experimental hurdles that need to be overcome for future progress. As an approach to reduce the seeding capacity of amyloid fibrils, the green tea polyphenol epigallocatechin gallate (EGCG) has a long history in protein aggregation research, as reviewed by Fernandes et al.. The authors summarize the effects of EGCG on PrP, huntingtin, α-synuclein and Aβ, including inhibition of aggregation, remodeling of fibrils to seeding-incompetent species, along with a reduction in neurotoxicity. Finally, Szegő et al. report the potential of the engineered binding protein β-wrapin AS69 to reduce fibril seeding in a PD mouse model. AS69 binds to the N-terminus of α-synuclein and interferes with fibril nucleation. When co-injected together with preformed fibril seeds (PFFs) into PD mice, AS69 inhibits PFF-induced α-synuclein pathology, suggesting that the α-synuclein N-terminus is a potential site for interference with aggregation and seeding.

## Author contributions

All authors listed have made a substantial, direct, and intellectual contribution to the work and approved it for publication.

